# MRI-Conditional Breast Tissue Expander: First In-Human Multi-Case Assessment of MRI-Related Complications and Image Quality

**DOI:** 10.3390/jcm12134410

**Published:** 2023-06-30

**Authors:** Simone Schiaffino, Andrea Cozzi, Barbara Pompei, Angela Lia Scarano, Carola Catanese, Armin Catic, Lorenzo Rossi, Filippo Del Grande, Yves Harder

**Affiliations:** 1 Imaging Institute of Southern Switzerland (IIMSI), Ente Ospedaliero Cantonale (EOC), 6900 Lugano, Switzerland; andrea.cozzi@eoc.ch (A.C.); angelalia.scarano@eoc.ch (A.L.S.); carolamialaura.catanese@eoc.ch (C.C.); filippo.delgrande@eoc.ch (F.D.G.); 2 Department of Plastic, Reconstructive and Aesthetic Surgery, Ente Ospedaliero Cantonale (EOC), 6900 Lugano, Switzerland; barbara.pompei@eoc.ch (B.P.); armin.catic@eoc.ch (A.C.); 3 Oncology Institute of Southern Switzerland (IOSI), Ente Ospedaliero Cantonale (EOC), 6500 Bellinzona, Switzerland; lorenzo.rossi@eoc.ch; 4 Breast Unit of Southern Switzerland (CSSI), Ente Ospedaliero Cantonale (EOC), 6500 Bellinzona, Switzerland; 5 Faculty of Biomedical Sciences, Università della Svizzera Italiana, 6900 Lugano, Switzerland

**Keywords:** mastectomy, breast reconstruction, breast tissue expander, magnetic resonance imaging, diffusion-weighted imaging, MRI safety, MRI-conditional, image quality, artifacts

## Abstract

This study aims to assess potential complications and effects on the magnetic resonance imaging (MRI) image quality of a new MRI-conditional breast tissue expander (Motiva Flora^®^) in its first in-human multi-case application. Twenty-four patients with 36 expanders underwent non-contrast breast MRI with T1-weighted, T2-weighted, and diffusion-weighted imaging (DWI) sequences on a 3 T unit before breast tissue expander exchange surgery, being monitored during and after MRI for potential complications. Three board-certified breast radiologists blindly and independently reviewed image quality using a four-level scale (“poor”, “sufficient”, “good”, and “excellent”), with inter-reader reliability being assessed with Kendall’s τ_b_. The maximum diameters of RFID-related artifacts on T1-weighted and DWI sequences were compared with the Wilcoxon signed-rank test. All 24 examinations were completed without patient-related or device-related complications. The T1-weighted and T2-weighted sequences of all the examinations had “excellent” image quality and a median 11 mm (IQR 9–12 mm) RFID artifact maximum diameter, significantly lower (*p* < 0.001) than on the DWI images (median 32.5 mm, IQR 28.5–34.5 mm). DWI quality was rated at least “good” in 63% of the examinations, with strong inter-reader reliability (Kendall’s τ_b_ 0.837, 95% CI 0.687–0.952). This first in-human study confirms the MRI-conditional profile of this new expander, which does not affect the image quality of T1-weighted and T2-weighted sequences and moderately affects DWI quality.

## 1. Introduction

Over the last decade, the number of patients undergoing mastectomies has continuously grown [1,2,3]. This phenomenon can be explained by several factors. The progressively easier access to genetic counseling has led to an increased number of patients with a confirmed genetic predisposition to breast cancer [4,5] who might eventually request a risk-reducing bilateral mastectomy [6,7,8]. Furthermore, the threshold for a mastectomy—particularly for high-risk patients—has steadily lowered, also because oftentimes patients preferring a mastectomy may avoid adjuvant radiotherapy of the residual glandular tissue. Finally, advancements in breast reconstruction techniques granting better and more durable results, both from the functional and aesthetic point of view [9,10], have contributed to the increase in patients’ desires for mastectomies, be they unilateral or bilateral, therapeutic or prophylactic [1,3,11,12].

Since breast cancer is by far the most common cancer in women [13,14], the high number of mastectomies has resulted in the largest group of patients requiring organ reconstruction in general and some form of breast reconstruction in particular [9,10,15]. This is confirmed by data from the US Agency for Healthcare Research and Quality, which demonstrate that during the 2009–2014 timeframe the rate of women getting breast reconstruction after a mastectomy increased from 21.7 to 35.1 per 100,000 [16]. To deal with this large number of women requiring breast reconstruction, two-stage “expander-to-implant” reconstruction is still the most common approach worldwide [16,17], accounting for approximately 60% of all breast reconstructions in 2020 [18].

Breast tissue expanders are implants that are inserted into a submuscular or subcutaneous pocket after a mastectomy, as most mastectomies are nowadays performed sparing the skin envelope of the breast [19,20]. Subsequently, usually in an outpatient setting, the expander is filled stepwise with sterile saline—as required—through a specific port [16]. Once the desired breast volume is achieved, the expander is then replaced with the definitive implant or with autologous tissue according to patient’s needs and wishes [21]. 

Breast tissue expanders, as many other medical devices, have been traditionally classified as “unsafe” for magnetic resonance imaging (MRI) [16]. In MRI, images are obtained through the interaction among the static magnetic field, the repeated switching of magnetic field gradients, and the radiofrequency pulses: these factors can interfere with any implanted device containing magnetic components, potentially harming the device and/or the patient [22]. While this aspect is of paramount importance for cardiac devices [23], breast tissue expanders also contain magnetically sensitive components in their filling port in order to guide its identification for periodic filling or deflation of the expander [24]. Indeed, although they are usually a temporary device, expanders may occasionally remain in place as long as 1–2 years, especially in patients undergoing adjuvant chemo- and radio-therapy and subsequent hybrid reconstruction of the breast, consisting of stepwise deflation of the expander and repeated grafting of autologous fat into the mastectomy flap before exchanging the expander for a definitive implant [25]. Nevertheless, these oncological patients with breast tissue expanders may require breast MRI to investigate potential surgical complications or for cancer surveillance during the first stage of breast reconstruction [26]. Likewise, these patients may also need to undergo MRI of other nearby organs for the evaluation of other comorbidities [16]. 

While several small studies reported that patients with MRI-unsafe breast tissue expanders could safely undergo breast MRI under selected conditions [27,28,29,30,31,32], patient discomfort, expander displacement, and MRI artifacts and signal loss still represent major issues [16].

In 2020, a comprehensive in vitro MRI study by Bayasgalan et al. [17] on a new magnet-free breast tissue expander (Motiva Flora^®^, Establishment Labs, Alajuela, Costa Rica) demonstrated that the expander achieved an MRI-conditional safety profile and that the small radio-frequency identification device (RFID) replacing the magnetic port was not damaged by MRI scanning. Shortly afterwards, this breast tissue expander was approved for use both by European and Swiss regulatory agencies.

This study presents the first in-human multi-case series of patients undergoing breast MRI after implantation of this new MRI-conditional breast tissue expander, aiming to assess potential complications and effects on overall MRI quality, with a focus on artifact-prone diffusion-weighted imaging (DWI) sequences.

## 2. Materials and Methods

### 2.1. Study Design and Population

This report is part of a larger controlled ambispective cohort study that aims to compare the surgical and clinical outcomes of patients undergoing a mastectomy and temporary reconstruction with a new MRI-conditional breast tissue expander versus those who have undergone the same surgery with other MRI-unsafe breast tissue expanders. The study was conducted at the Centro di Senologia della Svizzera Italiana (Ente Ospedaliero Cantonale, Lugano, Switzerland) after approval by the local ethics committee (Comitato Etico Cantonale, Bellinzona, Switzerland; protocol code: 2021-01916; reference CE 3954; approved on 30 December 2021). All enrolled patients signed the ad-hoc informed consent. 

The herein presented cohort includes all women enrolled from August 2020 to November 2022 who underwent a mastectomy followed by a two-stage breast reconstruction with the Motiva Flora^®^ breast tissue expander. All these women were referred for breast MRI during the time period between the implantation and removal of the tissue expander. 

All patients underwent breast MRI on a 3 T unit (MAGNETOM Skyra, Siemens Healthineers, Erlangen, Germany) with a dedicated eight-channel breast coil. At the time of imaging, the patients presented with variable levels of saline filling of the breast tissue expander according to clinical needs.

The non-contrast MRI protocol included axial turbo spin-echo T1-weighted Dixon sequences (TR 879 ms; TE 30 ms; flip angle 144°; slice thickness 3 mm), axial T2-weighted turbo inversion recovery magnitude (TIRM) sequences (TR 4980 ms; TE 72 ms; flip angle 80°; slice thickness 3 mm), and axial DWI sequences (b values 0, 1000 s/mm²); TR 11,800 ms; TE 66 ms; flip angle 180°; slice thickness 3 mm). 

Patients were monitored during MRI to evaluate the potential occurrence of immediate MRI- or expander-related complications, such as overheating, pain, or perceived sensation of expander displacement. After MRI, patients were interviewed about any discomfort they experienced during the examination and a plastic surgeon conducted a targeted physical examination with RFID testing to evaluate any potential expander displacement or any damage to the RFID port.

### 2.2. Image Quality Assessment

First, the image quality of all T1-weighted and T2-weighted sequences was assessed by a board-certified breast radiologist (Reader 1, S.S., with 6 years of experience in breast MRI), blinded to original reports and patients’ identifying information, using the institutional PACS viewer (Intellispace, Philips Healthcare, Best, The Netherlands). The following characteristics were assessed:

1. Overall image quality for diagnostic purposes, on a qualitative four-level Likert scale (“Poor”, “Sufficient”, “Good”, and “Excellent”);
2. Field homogeneity, with dichotomic assessment (“Affected” and “Not affected”);
3. Image noise, on a three-level ordinal scale (“Present and confounding interpretation”, “Present but not hindering interpretation”, and “Absent”);
4. Maximum diameter of the artifact caused by the RFID port on axial T1-weighted images (on the side with largest artifact in patients with bilateral expanders) and maximum diameter of the expander on the same axial plane, with calculation of their ratio.

Second, for DWI sequences, Reader 1 evaluated the following characteristics:

5. Overall image quality for diagnostic purposes, again on a four-level scale (“Poor”, “Sufficient”, “Good”, and “Excellent”);
6. Ability to distinguish thoracic anatomical structures (chest wall and sternum), with dichotomic assessment;
7. Quality of fat suppression, on a three-level ordinal scale (“Insufficient”, “Inhomogeneous”, and “Homogeneous”);
8. Presence of ghosting artifacts, on a three-level ordinal scale (“Present and confounding interpretation”, “Present but not hindering interpretation”, and “Absent”)
9. Maximum diameter of the RFID-related artifact.

Considering the inherent artifact-prone nature of DWI sequences, a second evaluation of the first four qualitative image characteristics was performed by two other certified breast radiologists (in consensus, Reader 2, A.L.S. and C.C., with 9 and 15 years of experience in breast MRI, respectively), blinded both to the original reports and to the results of the evaluation performed by Reader 1.

### 2.3. Statistical Analysis

Descriptive statistics were used to summarize data. Categorical variables were reported as numbers and percentages, whereas continuous variables were reported with median and interquartile range (IQR) or mean ± standard deviation according to their distribution, evaluated with the Shapiro–Wilk test. Inter-reader reliability for nominal dichotomous variables was estimated using Cohen’s κ, reported with 95% confidence intervals (Cis), and interpreted according to the Landis and Koch scale [33]. Inter-reader reliability for ordinal variables was estimated using Kendall’s τ_b_, reported with 95% CIs and interpreted as proposed by Le Roy [34]. The Wilcoxon signed-rank test was used to compare the artifact-to-expander ratios on the T1-weighted and DWI sequences. Analyses were conducted with SPSS (version 26.0, IBM Corp) and R (version 4.2.2, R Core Team), considering *p* values < 0.05 as statistically significant.

## 3. Results

A total of 24 MRI examinations were performed in 24 patients (median age 50 years, IQR 45–55), 12/24 (50%) with unilateral and 12/24 (50%) with bilateral expanders, for a total of 36 examined expanders.

All 24 MRI examinations were completed without any discomfort related to expander displacement or heating being reported by the patients. Physical examination with RFID testing conducted by a plastic surgeon after each of the 24 examinations did not reveal any expander displacement nor any post-procedural disfunction of the RFID ports of the 36 examined expanders.

**Figure 1:**
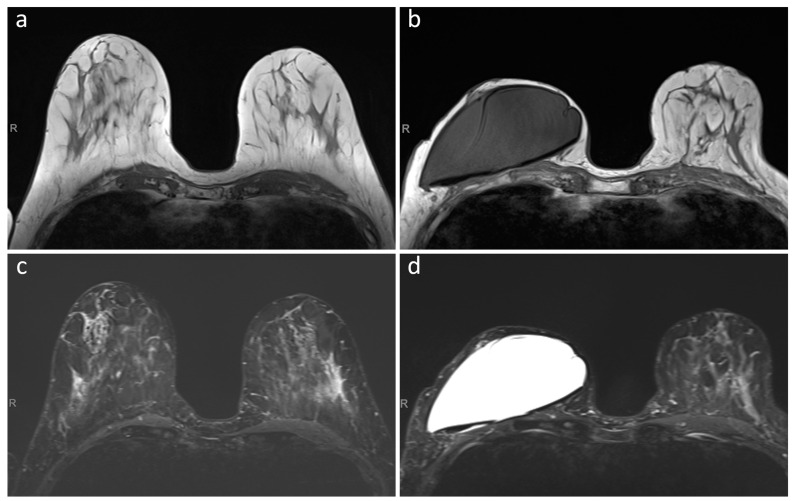
T1-weighted (panels (**a**,**b**)) and T2-weighted (panels (**c**,**d**)) MRI images of a patient that underwent a skin sparing mastectomy of the right breast with pre-pectoral implantation of the MRI-conditional breast tissue expander. (Panels (**a**,**c**)) show preoperative MRI images, while (panels (**b**,**d**)) show MRI images acquired prior to planned exchange surgery to definitive implant, with no negative effects on image quality caused by the presence of the tissue expander and its non-metallic port.

Blinded review of T1-weighted and T2-weighted images showed how these sequences remained unaffected by the presence of the expander, as also noted in the original MRI reports. In all 24 examinations, the highest T1-weighted and T2-weighted image quality was observed, and the presence of the expander neither influenced field homogeneity nor generated image noise (Figure 1). On T1-weighted images, the median maximum diameter of the RFID-related artifact on the axial plane was 11 mm (IQR 9.5–11.5 mm, range 4–14 mm) and the median maximum diameter of the breast tissue expander was 111 mm (IQR 104–118 mm), with a median ratio of 9.4% (IQR 8.1–11.0%) and no ratio higher than 14.1%.

**Figure 2:**
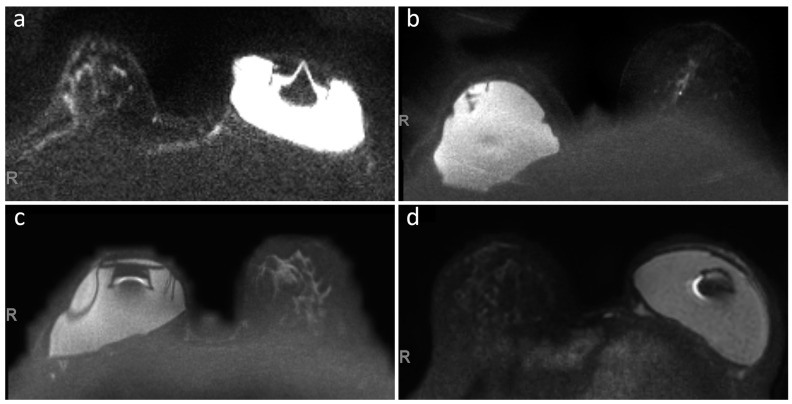
DWI images (*b* value 1000 s/mm²) of four patients showing different levels of overall image quality degradation caused by the presence of unilateral breast tissue expander, as rated by both readers. (**a**) “Poor” image quality”; (**b**) “Sufficient” image quality; (**c**) “Good” image quality; (**d**) “Excellent” image quality. Of note, in panels (**a**,**b**), the high field inhomogeneity makes the expander signal appear particularly hyperintense.

**Figure 3:**
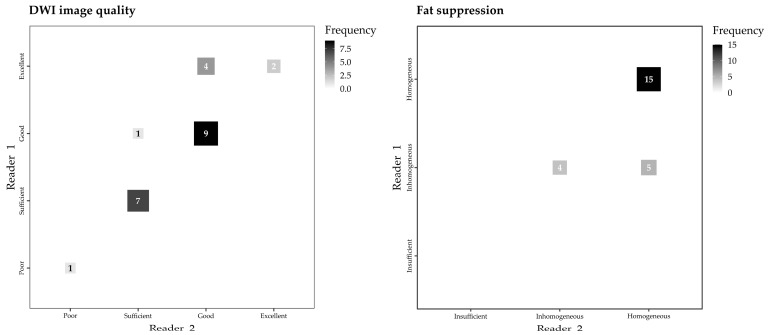
Fluctuation plots of Reader 1 and Reader 2 ratings of DWI image quality (Kendall’s τ_b_ 0.837, 95% CI 0.687–0.952) and fat suppression in DWI images (Kendall’s τ_b_ 0.577, 95% CI 0.295–0.852).

For DWI sequences, overall image quality (Figure 2) was graded “Poor” in 1/24 (4%) examinations by Reader 1 and in 1/24 (4%) examinations by Reader 2, “Sufficient” in 7/24 (29%) examinations by Reader 1 and in 8/24 (33%) examinations by Reader 2, “Good” in 10/24 (42%) examinations by Reader 1 and in 13/24 (55%) examinations by Reader 2, and “Excellent” in 6/24 examinations (25%) by Reader 1 and 2/24 examinations (8%) by Reader 2, with strong inter-reader reliability (Kendall’s τ_b_ 0.837, 95% CI 0.687–0.952, Figure 3). 

Perfect inter-reader reliability (Cohen’s κ 1.000, 95% CI 0.910–1.000) was observed for the evaluation of the thoracic anatomical features. According to both readers, the features were well distinguishable in 19/24 examinations (79%) and masked by artifacts in 5/24 examinations (21%). The presence of the breast tissue expander did not cause “Insufficient” fat suppression on DWI images in any of the 24 examinations, fat suppression being deemed at worst “Inhomogeneous” in 9/24 examinations by Reader 1 and in 4/24 examinations (17%) by Reader 2. Most examinations revealed “Homogeneous” fat suppression according to Reader 1 (15/24 examinations, 63%) and Reader 2 (20/24 examinations, 83%), with moderate inter-reader reliability (Kendall’s τ_b_ 0.577, 95% CI 0.295–0.852, Figure 3). 

**Figure 4:**
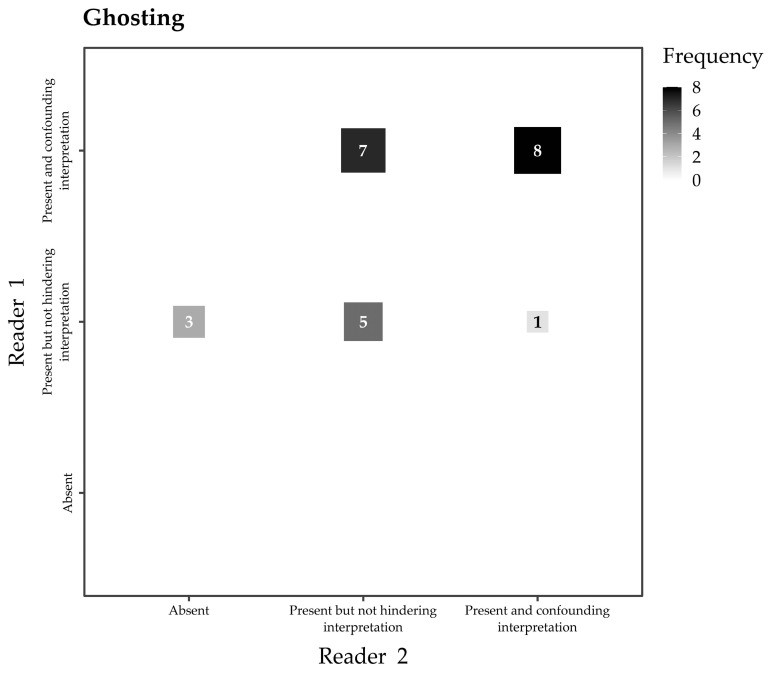
Fluctuation plot of Reader 1 and Reader 2 assessments of ghosting artifacts (Kendall’s τ_b_ 0.513, 95% CI 0.177–0.748).

Ghosting artifacts were deemed “Absent” in 15/24 examinations (63%) by Reader 1 and in 9/24 examinations (37%) by Reader 2, “Present but not hindering interpretation” in 9/24 examinations (37%) by Reader 1) and in 12/24 examinations (50%) by Reader 2, and “Present and confounding interpretation” in none of the examinations by Reader 1 and in 3/24 (13%) examinations by Reader 2, with moderate inter-reader reliability (Kendall’s τ_b_ 0.513, 95% CI 0.177–0.748, Figure 4). The maximum diameters of the RFID-related artifact (median 32.5 mm, IQR 28.5–34.5 mm) were significantly higher than those measured on T1-weighted images (z value 4.2, *p* < 0.001).

## 4. Discussion

Nowadays, as skin-sparing mastectomies are almost always offered when performing a mastectomy, some form of immediate breast reconstruction is required to benefit from the mastectomy pocket [19,20]. The vast majority of all breast reconstructions is still implant-based and accounts for 81% of all breast reconstructions in the US [18]. Implant-based breast reconstruction can be performed as a single-stage direct-to-implant placement (13% of total reconstructions) or as a two-stage expander-to-implant placement (68%) [18]. The high rate of expander-to-implant breast reconstruction is mainly due to many situations that do not allow or are disadvantageous for definitive direct-to-implant reconstruction of the breast. Accordingly, the use of breast tissue expanders is of paramount importance to bridge the time until definitive reconstruction [16,17,18], such as in the case of inadequate perfusion of the mastectomy flaps, in the indication for post-mastectomy radiotherapy, in some form of immediate–delayed autologous breast reconstruction due to fully-booked operating room capacities [35], and in undecided patients. Furthermore, breast tissue expanders are more and more often used in several hybrid approaches, i.e., the gradual deflation of the expander while injecting stepwise aspirated autologous fat into the mastectomy flaps, eventually resulting in fully autologous breast reconstruction [36] or in hybrid breast reconstruction, using if possible a down-sized definitive implant [25]. This recent trend might be influenced by the fact that autologous flap-based breast reconstruction is associated with better functional and aesthetic outcomes and improved durability compared to implant-based breast reconstruction, despite higher risks of both minor and major complications [37,38]. On the other hand, patients undergoing implant-based breast reconstructions are more likely to experience some form of reconstructive failure at mid-term, including rippling, rotation, malpositioning, capsular contracture, rupture, and extrusion which leads us to improve the quality and durability of implant-based breast reconstruction [39]. More recently, symptomatic or unappealing breast animation in sub-muscular implant-based breast reconstructions has also become an issue. Accordingly, placing the definitive implant whenever indicated in front of the pectoral muscle has become again more popular, because it is associated with superior clinical and functional and more favourable aesthetics outcomes compared to sub-muscular reconstruction [40]. This approach might improve the quality of implant-based breast reconstruction also in the long-term. Despite a certain trend towards pre-pectoral implant-based breast reconstruction, we feel that the use of synthetic meshes or acellular dermal matrices should be considered thoroughly and used in a very selected way [41,42]. Regardless of whether direct-to-implant breast reconstruction may be performed successfully, there will always be a large number of patients requiring breast-tissue expanders before undergoing definitive implant-based breast reconstruction.

Despite the major technological improvements of breast tissue expanders [43] the complication rate associated with their usage remains as high as 20%, including displacement, pain, capsular contracture, rupture, seroma formation, and infection [44]. While clinical examination and breast ultrasonography are sufficient in most cases, the use of MRI may be necessary in several unclear situations, as routinely employed in patients with silicone breast implants after definitive reconstruction [45]. Moreover, MRI as a precise oncological follow-up tool may become even more important in the future, as hybrid breast reconstruction will be offered more often and eventually breast tissue expanders will remain in place longer before exchange, mostly because repeated sessions of autologous fat grafting do take time and therefore extend the total length of stay of the expander. During the timeframe in which they have the expander in place, patients could need to undergo breast MRI for reasons unrelated to the evaluation of potential expander-related complications, e.g., for the assessment of metachronous symptomatic or non-symptomatic breast lesions detected during the follow-up in the ipsi- or contra-lateral breast [46]. Notably, the clinical management of these patients could also require an MRI examination of other organs to investigate secondary cancer, potentially also identified as collateral findings on the breast MRI performed after the insertion of the expander [47]. In these cases, the patients may be required to undergo abdominal MRI [48], brain MRI [49], and spinal MRI [50]. Furthermore, patients may also need to undergo cardiac MRI to assess myocardial damage caused by radiotherapy or chemotherapy [51].

Until the recent development of the first MRI-conditional breast tissue expander, the use of MRI frequently represented a clinical issue, as other breast tissue expanders were labelled MRI-unsafe due to the presence of magnetic components in the injection port. MRI of the breast, as well as of other nearby organs, was therefore contraindicated in these patients [16], with heavy repercussions on patient management. Accordingly, the off-label use of MRI in patients with MRI-unsafe expanders resulted not only in device-related complications such as pain, breast erythema, and expander displacement, but also in extensive image artifacts hindering MRI interpretation [16]. 

This study reports the first in-human multi-case application after skin sparing mastectomy of the Motiva Flora^®^ breast tissue expander—that had previously been labeled as MRI-conditional in an in vitro study by Bayasgalan et al. [17]—assessing potential MRI-related complications and effects on image quality. In 24 consecutive patients with 36 breast tissue expanders who underwent MRI before definitive breast reconstruction on a 3 T MRI unit, no MRI-related complications were reported during the examinations or observed at clinical examination performed by a plastic surgeon after MRI. Furthermore, all RFID ports of the expanders were correctly functioning when tested with the port locator, confirming the “MRI-conditional” profile of this breast tissue expander. 

**Figure 5:**
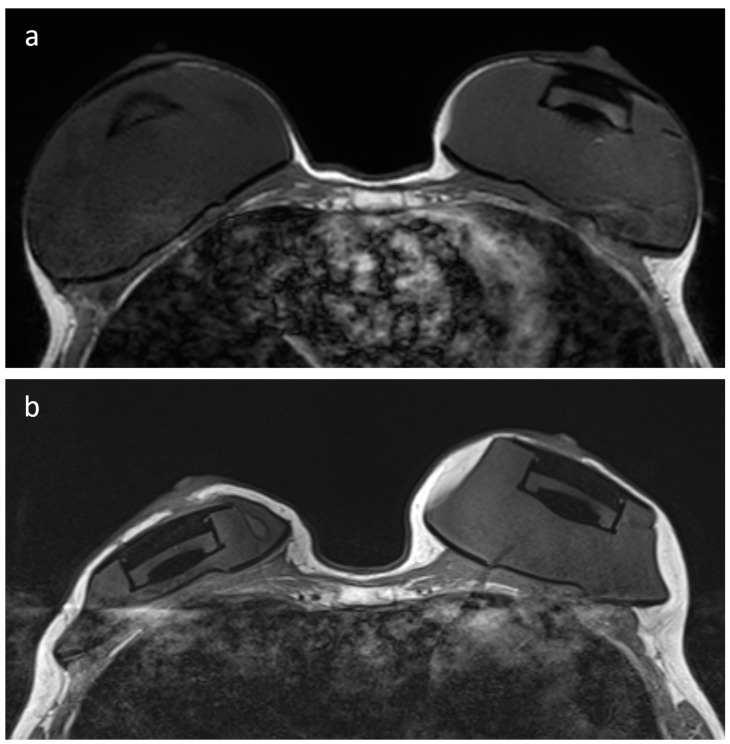
(**a**) T1-weighted Dixon in-phase image of a patient that underwent bilateral nipple sparing mastectomy and pre-pectoral implantation of MRI-conditional breast tissue expanders, showing two intact expanders in place. (**b**) MRI examination showing partial deflation of the right tissue expander after iatrogenic rupture. Even after substantial deflation of the tissue expander, the RFID-related artifact of the expander port does not impair the visualization of surrounding breast tissue, skin, and chest wall structures.

In 2016, Thimmappa et al. [29] demonstrated that patients with MRI-unsafe breast tissue expanders could undergo MRI of distant anatomical regions (e.g., abdomen and pelvis) on 1.5 T units without damage neither to the tissue nor to the device. However, these examinations were still heavily affected by expander-generated artifacts. Conversely, the current study proves that the image quality of the most important part of the breast MRI protocol—T1-weighted and T2-weighted sequences—was unaffected by the presence of the tissue expander. On T1-weighted images, no RFID-related artifact larger than 14 mm was observed. The small extension of these artifacts compared to the total volume of the breast tissue expander meant that no artifact extended outside the device, with a maximum 14.1% size ratio. Accordingly, visualization of potential residual breast tissue after mastectomy, of breast skin and of chest wall structures, was also unhindered. Of note, even when the overall diameter of the expander was reduced, due to device rupture before the examination (Figure 5), the RFID-related artifact did not affect image interpretation.

Even when considering DWI sequences, that are the most sensitive ones to magnetic susceptibility at higher field strengths, their overall image quality was graded as “Good” or “Excellent” in at least 63% of all examinations by both readers. Factors contributing to this less optimal image quality included the significantly higher maximum diameter of the RFID-related artifacts, inhomogeneous fat suppression, and the presence of ghosting artifacts in at least 37% of all examinations. Of note, among the patients included in this study, the combination of inhomogeneous fat suppression and ghosting artifacts determined a substantial limitation in the interpretation of DWI sequences only in one case. In all other cases, these image quality shortcomings did not constitute a substantial barrier to DWI interpretation and examination reporting. 

This study has some limitations. First, being the first multi-case experience with this breast tissue expander, it includes only a comparatively small number of patients that come from a single referral center. Moreover, the original case-control design of the primary study could not be replicated in this targeted sub-analysis, as patients with “MRI-unsafe” breast tissue expanders (i.e., patients in the control group) did not undergo MRI: therefore, readers were unable to blindly evaluate image quality in patients with other expander types, potentially overestimating the effects of the MRI-conditional expander on image quality. Finally, this study was conducted on a 3 T unit, which amplifies magnetic susceptibility issues of all sequences compared to units with less strong magnetic fields, such as the more commonly available 1.5 T MRI systems. While this represents a limitation from a purely methodological point of view, the fact that T1-weighted and T2-weighted sequences were substantially unaffected by the presence of the expander and that DWI sequences were affected in a higher but still limited way makes it likely that examinations performed on units with lower field strengths will be even less affected by the presence of this MRI-conditional breast tissue expander.

## 5. Conclusions

In this first in-human multi-case series of 3 T breast MRI examinations performed on patients with a MRI-conditional breast tissue expander, neither MRI-related complications nor MRI-related damage to the expander RFID port were observed. T1-weighted Dixon sequences and T2-weighted TIRM sequences were not affected by the presence of the breast tissue expander, while image quality of DWI sequences was moderately affected in about 40% of all cases.

## Data Availability

Data analyzed in this study are contained within the article. Requests for data from the primary study can be addressed to the corresponding author and will be subjected to institutional and ethical approval according to national and local regulations on data sharing and transfer.
